# Functional and Genetic Landscape of Complement Dysregulation Along the Spectrum of Thrombotic Microangiopathy and its Potential Implications on Clinical Outcomes

**DOI:** 10.1016/j.ekir.2021.01.034

**Published:** 2021-02-03

**Authors:** Sjoerd A.M.E.G. Timmermans, Jan G.M.C. Damoiseaux, Alexis Werion, Chris P. Reutelingsperger, Johann Morelle, Pieter van Paassen

**Affiliations:** 1 Department of Nephrology and Clinical Immunology; 3 Central Diagnostic Laboratory, Maastricht University Medical Center, Maastricht, The Netherlands; 2 Department of Biochemistry, Cardiovascular Research Institute, Maastricht, The Netherlands; 4 Division of Nephrology, Cliniques Universitaires Saint-Luc, Brussels, Belgium; 5 Institut de Recherche Experimentale et Clinique, UCLouvain, Brussels, Belgium

**Keywords:** atypical hemolytic uremic syndrome, complement, eculizumab, hypertensive emergency, kidney transplantation, pregnancy, thrombotic microangiopathy

## Abstract

**Introduction:**

The syndromes of thrombotic microangiopathy (TMA) are diverse and represent severe endothelial damage caused by various mechanisms. The complement system plays a major role in a subset of patients with TMA, and its recognition is of clinical importance because it guides choice and duration of treatment.

**Methods:**

We studied a well-defined cohort of patients with TMA and hypothesized that assessment of serum-induced *ex* *vivo* C5b9 formation on the endothelium and screening for rare variants in complement genes can better categorize TMA.

**Results:**

Massive *ex vivo* C5b9 formation was found in all patients with primary atypical hemolytic uremic syndrome (*n*/*N* = 11/11) and in 59% of patients with TMA and coexisting conditions (*n*/*N* = 30/51). Massive *ex vivo* C5b9 formation was associated with rare genetic variants (45% [*n*/*N* = 20/44] vs. 0% [*n*/*N* = 0/21] patients with normal *ex vivo* C5b9 formation; *P* < 0.001). Massive *ex vivo* C5b9 formation was associated with favorable renal response to therapeutic complement inhibition in patients with TMA and coexisting conditions (86% [*n*/*N* = 12/14] vs. 31% [*n*/*N* = 5/16] of untreated patients; *P* < 0.001), indicating complement-mediated TMA rather than secondary disease. Among treated patients, the odds ratio for 1-year kidney survival was 12.0 (95% confidence interval 1.2-115.4). TMA recurrence was linked to rare genetic variants in all cases. Patients with normal *ex vivo* C5b9 formation had an acute, nonrelapsing form of TMA.

**Conclusions:**

*Ex vivo* C5b9 formation and genetic testing appears to categorize TMAs into different groups because it identifies complement as a driving factor of disease, with potential therapeutic and prognostic implications.

## Graphical abstract

**Figure:**
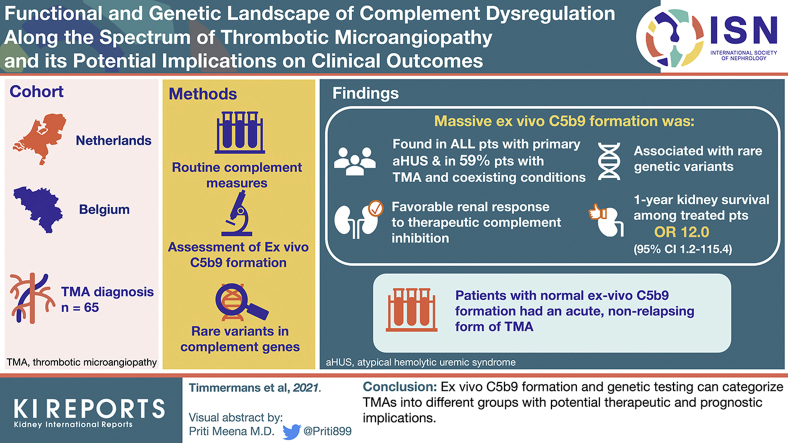

The syndromes of TMA are diverse and represent tissue responses to severe endothelial damage caused by various mechanisms.^1^ TMAs translate into microvascular thrombosis, thrombocytopenia, microangiopathic hemolysis, and ischemic organ damage, often affecting the kidneys. Complement dysregulation related to rare variants in complement genes or autoantibodies that interfere with complement regulation is a major risk factor for TMA in a subset of patients,^2^^,^^3^ referred to as complement-mediated TMA (C-TMA). Ever since the approval of therapeutic complement inhibition,^4^, ^5^, ^6^ the approach of the TMAs has transformed, focusing on the recognition of complement dysregulation in the earliest possible stage of disease.^7^^,^^8^

C-TMA should be considered after the exclusion of other well-established causes of TMA that are unrelated to complement dysregulation, such as thrombotic thrombocytopenic purpura and Shiga toxin–producing *Escherichia coli* infection.^9^ The diagnosis of C-TMA is challenging because reliable tests are lacking and, according to the current nomenclature, should be reserved for patients not presenting with coexisting conditions (i.e., primary atypical hemolytic uremic syndrome [aHUS]).^7^^,^^8^ Many such patients, however, require a coexisting condition, such as hypertension, to lower the threshold for C-TMA.^10^^,^^11^ Recently, we showed that massive *ex vivo* C5b9 formation on the endothelium indicates C-TMA in patients with coexisting hypertensive emergency, pointing to complement dysregulation rather than hypertension as the cause of TMA.^12^^,^^13^ C-TMA, in particular, was common in patients not responding to standard of care (i.e., blood pressure control), with high rates of progression to end-stage kidney disease (ESKD).^13^

We hypothesized that the prevalence of C-TMA is underappreciated in patients presenting with coexisting conditions beyond hypertensive emergency and that the assessment of both *ex vivo* C5b9 formation and screening for rare variants in complement genes better categorizes patients along the spectrum of TMA into different groups, with potential therapeutic and prognostic implications. We tested this premise in a well-defined cohort of 65 patients with TMA, either with or without coexisting conditions, and severe kidney involvement, often confirmed after obtaining a biopsy specimen of the kidney. Furthermore, the dynamics of *ex vivo* C5b9 formation in patients treated with therapeutic complement inhibition and a prolonged interdose interval were studied.

## Methods

### Patient Population and Definitions

Patients with TMA were recruited from the Limburg Renal Registry, Maastricht, The Netherlands,^14^ and the Cliniques universitaires Saint-Luc, Brussels, Belgium. TMA was defined as typical morphologic features of TMA on a biopsy specimen of the kidney or the triad of microangiopathic hemolytic anemia (hematocrit <30%, hemoglobin <10 g/l, lactate dehydrogenase >500 U/l, and schistocytes on peripheral blood smear), platelets <150 × 10^9^/l, and acute kidney injury. Patients presenting with coexisting conditions were classified as secondary aHUS according to HUS International’s nomenclature (Supplementary Methods).^7^^,^^8^ Patients with thrombotic thrombocytopenic purpura, defined as an enzymatic activity of von Willebrand cleaving protease <10% or the combination of platelets <30 × 10^9^/l and serum creatinine ≤200 μmol/l,^15^ and those with a Shiga toxin–producing *E coli* infection were excluded.

Clinical and laboratory data were documented at the time of presentation and during follow-up. The information was specified in the Limburg Renal registry and the patient’s medical records. Complete renal remission (CR) was defined as the restoration of an estimated glomerular filtration >60 ml/min/1.73 m^2^; partial renal remission (PR) was defined as the recovery of kidney function after dialysis or a >25% decrease in serum creatinine. The stage of chronic kidney disease (CKD) was based on international consensus^16^; ESKD was defined as the need for chronic kidney replacement therapy.

At the time of presentation and during follow-up, serum samples were obtained, processed, and immediately stored at −80 °C to prevent *in vitro* complement activation.^17^ The study was approved by the appropriate ethics committees and is in accordance with the Declaration of Helsinki.

### Routine Complement Measures

C4 and C3 serum levels and the functional activity of the classical pathway (Svar Life Sciences, Malmo, Sweden) were assessed.

### *Ex Vivo* C5b9 Formation on the Endothelium

*Ex vivo* C5b9 formation on microvascular endothelial cells of dermal origin (i.e., human microvascular endothelial cell-1 [HMEC-1]; ATCC, Manassas, Virginia, USA) was assessed as described.^12^ Briefly, HMEC-1 cells were plated on glass culture slides and used when >80% confluent, incubated with serum diluted in test medium for 3 hours at 37 °C, fixed in 3% formaldehyde, and blocked with 2% bovine serum albumin for 1 hour. The results of 26 patients have been published.^13^ In selected experiments, HMEC-1 cells were preincubated with 10 μM adenosine diphosphate for 10 minutes to mimic a perturbed endothelium.^18^ Rabbit anti-C5b9 pAb (Calbiochem, San Diego, California, USA) and Alexa488 labeled goat anti-rabbit Ab (Life Technologies, Carlsbad, California, USA) were used. Fluorescent staining was acquired in 15 fields and the staining area was evaluated using ImageJ software (National Institutes of Health, Bethesda, Maryland, USA). The samples were compared with pooled serum from 10 healthy control subjects run in parallel; *ex vivo* C5b9 formation on the resting and perturbed endothelium did not differ between individuals (data not shown).

### Rare Variants in Complement Genes and Factor H Autoantibodies

Patients were screened for rare variants, i.e., variants with a minor allele frequency <0.1%, and single-nucleotide polymorphisms in *CFH*, *CFI*, *CD46*, *CFB*, *C3*, *CFHR1*, *CFHR2*, *CFHR3*, *CFHR4*, *CFHR5*, *THBD*, and *DGKE* using DNA sequencing. The results of 49 patients have been published.^19^ The classification of variants was based on international standards.^20^ Pathogenic variants were defined as those with functional studies supporting a defect in complement regulation, including null variants in genes linked to complement regulation, variants located in a mutational hotspot, variants located in a functional domain, or variants that cluster in patients with primary aHUS as demonstrated by Osborne *et al.*^21^ Rare variants not fulfilling these criteria have been classified as uncertain significance.

Rearrangements in the *CFH-CFHR1-5* genomic region were analyzed by multiplex-ligation probe amplification. In selected cases, the presence of factor H autoantibodies (FHAA) was assessed by enzyme-linked immunosorbent assay.^22^

### Statistics

Continuous variables were presented as mean (± standard deviation [SD]) or median (interquartile range [IQR]) as appropriate. Differences in continuous and categorical variables were checked using the unpaired *t* or Mann-Whitney *U* tests and the χ^2^ or Fisher exact tests, respectively. *Ex vivo* C5b9 formation on the endothelium was compared with normal human serum run in parallel by the paired sample *t* test or Wilcoxon signed rank test as appropriate. Logistic regression was used to compute an odds ratio with 2-sided 95% confidence interval. Survival was assessed using the Kaplan-Meier methods and log-rank test.

Massive *ex vivo* C5b9 formation or pathogenic variants in complement genes defined C-TMA.

## Results

### Patient Population

Ninety-three patients with TMA were recruited (Supplementary Figure S1). Fifteen patients with acquired thrombotic thrombocytopenic purpura and 13 patients with antiphospholipid syndrome–related TMA, described previously,^23^ were excluded; serum samples obtained at the time of presentation from 14 of these patients were used as disease controls for *ex vivo* C5b9 formation. Thus, 65 patients with TMA were included (Table 1); 59 patients were of European descent, 4 patients of African descent, 1 patient of Latin American descent, and 1 patient of Asian descent. Patients invariably presented with severe kidney involvement, including 38 (58%) patients who initially needed dialysis. Microangiopathic hemolysis, low platelets, or both were present in 10 (15%), 16 (25%), and 26 (40%) cases. TMA was confirmed on a kidney biopsy specimen in 47 (72%) cases, including 39 patients not presenting with systemic hemolysis. Low levels of C4 and C3, measured at the time of presentation, were found in 5 (*N* = 57, 9%) and 19 (*N* = 59, 32%) patients, respectively. Fifty-two (80%) patients presented with coexisting conditions and should have been classified as secondary aHUS according to HUS International’s nomenclature.

**Table 1:** Main clinical data of 65 patients with TMA

	C-TMA	Normal complement regulation	*P* value
Patients, *n*	44	21	
HUS International’s nomenclature,^7^*n* (%)			
Primary aHUS	13 (30)	0	0.006
Secondary aHUS	31 (70)	21 (100)	0.006
Hypertensive emergency	18	12	
Pregnancy	8	0	
TMA after kidney transplantation	2	3	
Postsurgical TMA	2	1	
Streptococcal HUS	1	0	
HELLP	0	3	
Drug-induced TMA	0	2	
Features at presentation			
M/F, *n*	19/25	12/9	0.4
European, *n* (%)	43 (98)	16 (76)	0.01
Age, yr, mean ± SD	36±18	42±13	0.1
Creatinine, μmol/L, median (IQR)	492 (314–804)	485 (231–778)	0.5
Dialysis, *n* (%)	27 (61)	11 (52)	0.6
Hemolysis, *n* (%)	25 (57)	11 (52)	0.8
Systemic hemolysis, *n* (%)	18 (41)	8 (38)	1.0
Platelets, ×10^9^/l, median (IQR)	101 (44–228)	95 (52–178)	0.8
LDH, U/l, median (IQR)	842 (398–2103)	762 (465–1222)	0.6
ADAMTS13 activity >10%, *n*/*N*	31/31	17/17	
Low C4, *n*/*N*	5/39	0/18	0.2
Low C3, *n*/*N*	18/41	1/18	0.005
Massive ex vivo C5b9 formation, *n*/*N*	41/41	0/21	<0.001
Rare variant(s)/FHAA, *n* (%)	20 (45)	0 (0)	<0.001
Pathogenic, *n* (%)	17 (37)	0 (0)	0.006
Combined variants, *n*	2	0	1.0
MCPggaac, *n*/*N*	16/31	12/19	0.6
Treatment			
Plasma therapy, *n* (%)	31 (70)	7 (33)	0.007
Immunosuppression, *n* (%)	12 (27)	2 (10)	0.1
Eculizumab, *n* (%)	19 (43)	5 (26)	0.2
Days after diagnosis, median (range)	6 (0–100)	4 (2–37)	0.8
Doses, median (range)	13 (2–70)	4 (1–10)	0.009
Ongoing, *n*/*N*	3/19	0/6	0.6
Clinical outcome			
Patients, *n*/*N*	43/44	20/21	
Follow-up, yr, median (IQR)	2.0 (0.6–3.8)	0.5 (0.3–2.4)	0.002
Renal response, *n* (%)	24 (56)	9 (45)	0.6
Complete remission, *n*	15	4	0.4
Partial remission, *n*	9	5	0.8
ESKD at 3 months, *n* (%)	17 (40)	7 (35)	0.8
ESKD at last follow-up, *n* (%)	19 (44)	8 (45)	0.8
Patients with TMA recurrence, *n* (%)	11 (26)	0	0.01
Deceased at 3 months, *n* (%)	1 (2)	1 (5)	0.5
Deceased at last follow-up, *n* (%)	3 (7)	2 (10)	0.6

ADAMTS13, a disintegrin and metalloproteinase with a thrombospondin type 1 motif, member 13 (also known as von Willebrand factor cleaving protease); aHUS, atypical hemolytic uremic syndrome; C-TMA, complement-mediated thrombotic microangiopathy; ESKD, end-stage kidney disease; F, female; FHAA, factor H autoantibodies; HELLP, hemolysis, elevated liver enzymes, low platelets; IQR, interquartile range; LDH, lactate dehydrogenase; M, male; MCPggaac, at-risk haplotype for C-TMA; TMA, thrombotic microangiopathy.

Patients with *de novo* TMA after kidney transplantation presented with ESKD related to glomerular disease (antineutrophil cytoplasmic antibody–associated glomerulonephritis, *n* = 1; immunoglobulin A nephropathy, *n* = 1; and focal segmental glomerulosclerosis, *n* = 1), renovascular disease (*n* = 1), and reflux nephropathy (*n* = 1) in their native kidneys.

### Complement Workup

**Figure 1:**
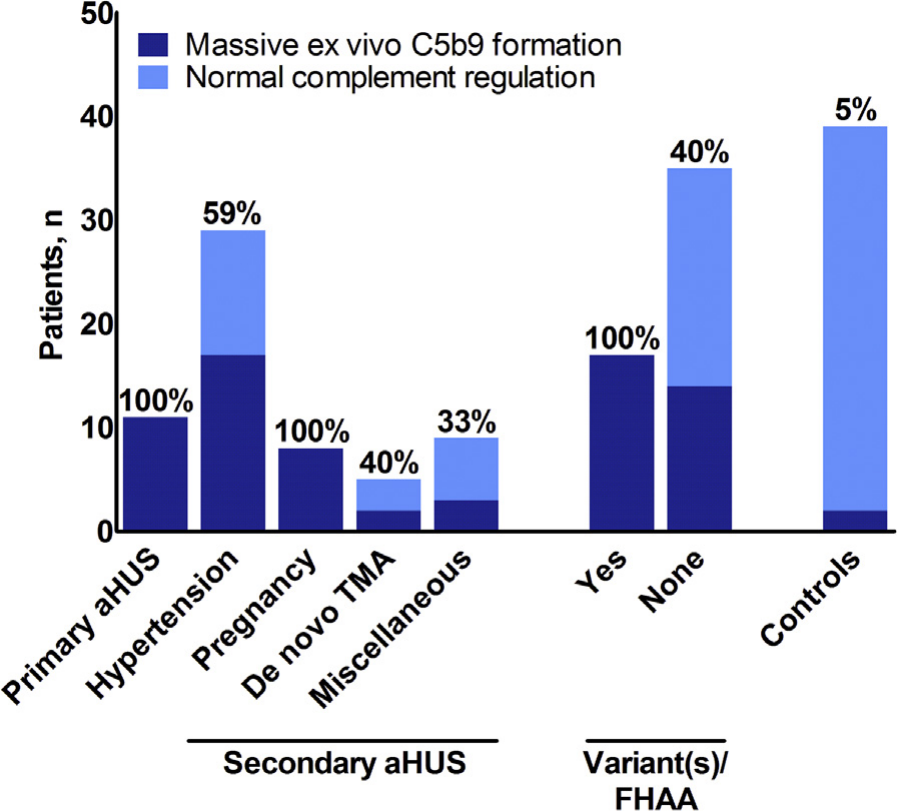
Massive *ex vivo* C5b9 formation along the spectrum of thrombotic microangiopathy (TMA) on the resting endothelium. Control subjects have been tested on the perturbed endothelium (Supplementary Table S4). De novo TMA indicates kidney donor recipients. aHUS, atypical hemolytic uremic syndrome; FHAA, factor H autoantibodies.

Patients were screened for complement dysregulation using functional (Figure 1) and genetic tests (Table 2); treatment-naïve serum samples from 62 of 65 (95%) patients with TMA (coexisting conditions, *n* = 51; primary aHUS, *n* = 11) were tested for *ex vivo* C5b9 formation at the time of presentation. No baseline serum sample was available from 3 patients with pathogenic variants in complement genes.

**Table 2:** The phenotype of patients carrying rare variants (MAF <0.1%) in complement genes or FHAAs^a^

Case no.	Sex/age, yr	Creatinine, μmol/L	MAHA	Platelets, ×10^9^/L	Treatment	Outcome	Gene/FHAA	Variant	Protein	MAF, %	*In vitro*	Significance
C-TMA and no coexisting conditions (i.e., primary aHUS)												
M00018	F/3	92	+	12	PEX, Ecu	CR, Rec.	C3	c.481C>T	R161W	<0.01	GOF^47^	Pathogenic
							*CFI*	c.392T>G	L131R	<0.01	LOF^48^	Pathogenic
M11317	M/65	372	+	44	PEX	CR	*CFHR5*	c.1412G>A	G471E	<0.07	Unknown	VUS
M00016	M/4	311	+	28	PEX, CS	CR	FHAA	c.CFHR1/3 del	p.CFHR1/3 del	N/A	N/A	N/A
M01609	M/20	287	+	345	PEX	CR	C3	c.481C>T	R161W	<0.01	GOF^47^	Pathogenic
M03103	F/12	339	+	264	PEX	ESKD, Rec.	C3	c.481C>T	R161W	<0.01	GOF^47^	Pathogenic
M00004	F/49	800	+	<150	PEX	ESKD, Rec.	*CFI*	c.1420C>T	R474∗	<0.01	LOF^49^	Pathogenic
B12	F/31	359	–	23	PEX, Ecu	CR	*CFH*	c.3486delA	K1162Nfs∗7	0	Unknown	Pathogenic
B27	M/53	441	+	53	PEX, Ecu	CKD G3b	CD46	c.478G>T	V160F	0	Unknown	VUS
B39	M/25	469	+	7	PEX	CR	C3	c.3125G>A	R1042Q	0	Unknown	VUS
C–TMA and coexisting hypertensive emergency												
M99917	M/47	1980	–	272	Ecu	CKD G3b	*CFI*	c.148C>G	P50A	<0.02	LOF^50^	Pathogenic
							*THBD*	c.1433C>T	T478I	<0.01	Unknown	VUS
M02715	F/28	1065	–	228	PEX	ESKD	C3	c.481C>T	R161W	<0.01	GOF^47^	Pathogenic
M01715	F/41	334	–	291	PEX, Ecu	CKD G4	*CFI*	c.452A>G	N151S	<0.01	LOF^50^	Pathogenic
M04010	F/32	1138	+	142	PEX	ESKD, Rec.	*CFH*	c.2558G>A	C853Y	0	LOF^51^	Pathogenic
M03307	M/37	586	+	100	PEX	ESKD, Rec.	C3	c.481C>T	R161W	<0.01	GOF^47^	Pathogenic
M04306	M/40	1195	+	158	PEX	ESKD, Rec.	CD46	c.811_816delGACAT	D271/S272	0	LOF^52^	Pathogenic
M00105	F/38	1730	+	228	–	ESKD, Rec.	C3	c.481C>T	R161W	<0.01	GOF^47^	Pathogenic
M05486	M/39	1089	–	101	–	ESKD, Rec.	C3	c.481C>T	R161W	<0.01	GOF^47^	Pathogenic
C-TMA and coexisting pregnancy (i.e., pregnancy-associated atypical HUS)												
M00503	F/32	1388	+	212	PEX, CS	ESKD, Rec.	C3	c.481C>T	R161W	<0.01	GOF^47^	Pathogenic
B46	F/31	557	+	77	PEX, Ecu	ESKD	*CFI*	c.772G>A	A258T	0.01-0.03	LOF^48^	Pathogenic
C-TMA and coexisting kidney transplantation (i.e., *de novo* TMA after kidney transplantation)												
B33	M/24	309	–	252	PEX, Ecu	CKD G4/T	*CFI*	c.148C>G	P50A	<0.02	LOF^50^	Pathogenic

aHUS, atypical hemolytic uremic syndrome; CKD, chronic kidney disease; CR, complete renal remission; CS, corticosteroids; Ecu, eculizumab; ESKD, end-stage kidney disease; F, female; FHAA, factor H autoantibody; GOF, gain of function; LOF, loss of function; M, male; MAF, minor allele frequency in the European American population according to the Exome Variant Server and Genome Aggregation Database; MAHA, microangiopathic hemolytic anemia; PEX, plasma therapy; Rec, recurrence; TMA, thrombotic microangiopathy; VUS, variant of uncertain significance.

Case numbers beginning with a B are from the Brussels cohort, and case numbers beginning with an M are from the Maastricht cohort.

a Rare variants in complement genes/FHAAs were found in 9 of 13 (69%) patients with primary aHUS, 8 of 30 (27%) patients with coexisting hypertensive emergency, 2 of 8 (25%) patients with coexisting pregnancy, and 1 (20%) patient with *de novo* TMA after kidney transplantation.

#### C-TMA

Massive *ex vivo* C5b9 formation on the resting endothelium was found in 41 of 62 (66%) patients tested for, including 30 of 51 (59%) patients with coexisting conditions. This was particularly the case in patients with coexisting hypertensive emergency (*n*/*N* = 17/29, 59%), pregnancy (*n*/*N* = 8/8, 100%), and de novo TMA after kidney transplantation (*n*/*N* = 2/5, 40%). At the time of quiescent disease, *ex vivo* C5b9 formation normalized on the resting (*n*/*N* = 12/12, 100%) but not perturbed endothelium (*n*/*N* = 7/9, 78%) when using samples from patients not treated with therapeutic complement inhibition who had massive *ex vivo* C5b9 formation at the time of acute TMA. Thus, massive *ex vivo* C5b9 formation is not a secondary phenomenon triggered by acute TMA (e.g., hemolysis^24^ and fibrinolysis^25^).

Ten rare variants in complement genes were identified in 17 of 41 (41%) patients with massive *ex vivo* C5b9 formation (Table 2, Supplementary Table S1); 7 variants were considered pathogenic and 3 were of uncertain significance. The genomic deletion of *CFHR1* and *CFHR3* was found in homozygosity in 3 patients and associated with FHAA in 1 case, i.e., deficiency of CFHR plasma proteins and autoantibody positive (DEAP) HUS. In addition, 3 patients not tested for *ex vivo* C5b9 formation had pathogenic variants identified (i.e., M99917, M00004, and B03), 1 of whom also carried 1 variant of uncertain significance. In total, 44 patients had C-TMA. Two of 44 (5%) patients presented with combined variants.

#### Normal Complement Regulation

Normal *ex vivo* C5b9 formation on both the resting and perturbed endothelium was found in 21 patients with coexisting conditions, indicating true secondary aHUS with normal complement regulation.^18^ The specificity of normal *ex vivo* C5b9 formation on the perturbed endothelium for normal complement regulation is 95% as based on 39 control samples (Figure 1, Supplementary Table S2). Low C3 levels were found in 1 of 18 patients tested for, contrasting patients with C-TMA (*n*/*N* = 18/41, *P* = 0.005). None of the patients with normal complement regulation carried rare variants in complement genes or FHAA. Thus, none of the patients with normal *ex vivo* C5b9 formation had identified genetic or acquired abnormalities associated with complement dysregulation.

### The Clinical Course of TMA

The clinical data of 65 patients with TMA classified according to HUS International’s nomenclature are shown in Supplementary Table S1.

#### C-TMA and Coexisting Conditions

Thirty-one of 51 (61%) patients with coexisting conditions had C-TMA rather than secondary aHUS. These patients presented with coexisting hypertensive emergency (*n* = 18), pregnancy (*n* = 8), de novo TMA after kidney transplantation (*n* = 2), postsurgical TMA (*n* = 2), or streptococcal HUS (*n* = 1); rare variants in complement genes confirmed the genetic predisposition in 11 patients (Table 2).

Of the patients with C-TMA and coexisting conditions, 30 had follow-up data available, with a median follow-up of 2.3 years (IQR 0.7–6.5 years; Table 1). Twenty-one of 30 (70%) patients were treated with plasma therapy. Fourteen patients not responding to standard of care, including plasma therapy in 12 patients, were treated with eculizumab (Supplementary Table S3); treatment was initiated after a median of 7 days (range 1–100 days) and a median of 14 doses (range 4–70 doses) were administered. In addition, 11 patients were treated with immunosuppressive drugs, including 2 kidney donor recipients (i.e., prophylactic treatment for rejection; Supplementary Table S4).

**Figure 2:**
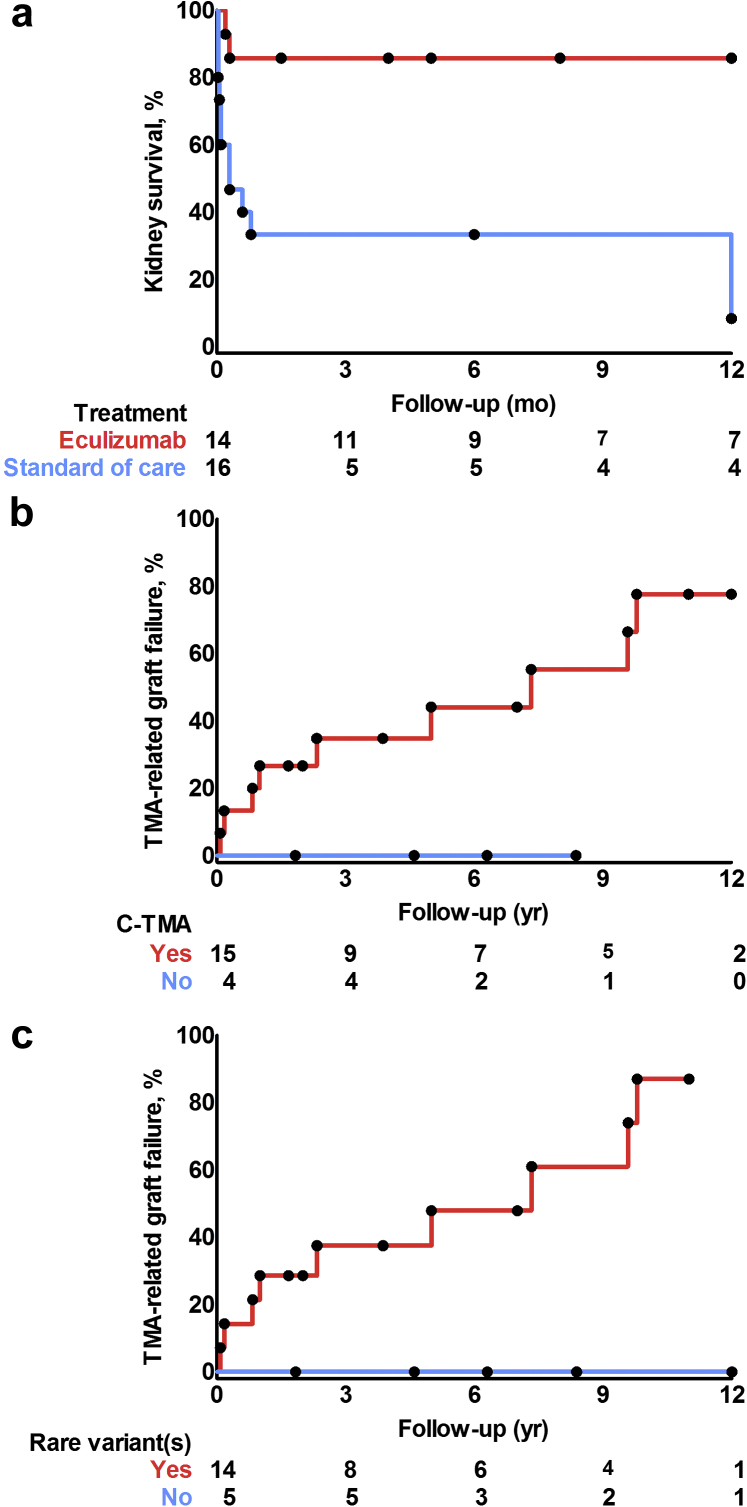
Kaplan-Meier curves. (a) End-stage kidney disease was less prevalent in patients with complement-mediated thrombotic microangiopathy (C-TMA) and coexisting conditions who had been treated with eculizumab compared with untreated patients (log-rank test, *P* < 0.001). (b) TMA recurrence after kidney transplantation was common in patients with C-TMA and (c) linked to rare variants in complement genes (log-rank test, *P* < 0.05).

In 14 of 30 (47%) patients, a renal response was achieved, either a CR (*n* = 6) or PR (*n* = 8). The cumulative incidence of renal response did not differ between patients with rare variants in complement genes and those with no variants identified (3/11 vs. 11/19 patients, *P* = 0.1). PR was associated with CKD stage G3 to G4 in 7 patients and G5 in 1 patient at 3 months; the patient with CKD stage G5 improved to G4 at 1 year. Renal response rates were higher in patients treated with eculizumab compared with untreated patients (Figure 2a), as discussed later. Of note, 13 of 14 (93%) remitted patients (i.e., those patients who achieved a renal response) had a sustained clinical remission during follow-up. The patient with no sustained clinical remission presented with streptococcal HUS recurrence in the native kidneys, similar to the first event—that is, linked to massive *ex vivo* C5b9 formation and a CR upon a 3-month course of eculizumab.

The cumulative incidence of ESKD was 16 (53%), including 15 patients who progressed to ESKD within 3 months. ESKD did not differ between patients with rare variants in complement genes and those with no variants identified (8/11 vs. 8/19 patients, *P* = 0.1). Eculizumab was associated with better 1-year kidney survival as 12 of 14 (86%) treated patients achieved a renal response, whereas 11 of 16 (69%) untreated patients progressed to ESKD within 3 months (Figure 2a); baseline characteristics are shown in Supplementary Table S3. The odds ratio for 1-year kidney survival was 12.0 (95% confidence interval 1.2–115.4) when treated with eculizumab. Of note, the nonresponding patients presented either with anuric or oliguric kidney disease, serum creatinine >550 μmol/l, and severe interstitial fibrosis/tubular atrophy on review of the kidney biopsy specimen (i.e., >50%).

Thirteen donor kidneys were transplanted in 8 recipients, including 7 patients with pathogenic variants in complement genes. Most kidneys were transplanted before the approval of eculizumab by the European Medicines Agency in 2011. Preemptive eculizumab was initiated in 1 patient who carried a pathogenic variant in *CFH* and prevented graft failure for ≥7 years. Ten episodes of TMA recurrence were documented in 9 donor kidneys from 7 recipients, and all but 1 patient carried pathogenic variants in complement genes. TMA recurrence resulted in graft loss in all but 1 case (Figures 2b and 2c). Of note, 1 recipient with advanced graft failure (serum creatinine 486 μmol/l) on the background of chronic TMA lost his donor kidney after 12 months despite eculizumab.^26^^,^^27^

Three patients died during follow-up; the causes of death were cardiac disease (*n* = 2) and cancer (*n* = 1).

#### C-TMA and No Coexisting Conditions (i.e., Primary aHUS)

Most patients with C-TMA classified as primary aHUS presented with profound systemic hemolysis and less severe kidney disease compared with those with C-TMA and coexisting conditions (Supplementary Table S1). The prevalence of rare variants in complement genes did not differ from patients with C-TMA and coexisting conditions.

Follow-up data were available for all patients, with a median follow-up of 2.1 years (IQR 1.0–9.1 years). Ten of 13 (77%) patients were treated with plasma therapy. Eculizumab was initiated in 5 patients. The patient with DEAP-HUS was treated with steroids.

Ten of 13 (77%) patients achieved a renal response, either a CR (*n* = 9) or PR (*n* = 1). The cumulative incidence of renal response did not differ from patients with C-TMA and coexisting conditions, whereas ESKD at 3 months appeared more common in the latter. Three donor kidneys were transplanted in 2 recipients with pathogenic variants; TMA recurrence and subsequent graft failure were documented in each case (Figures 2b and 2c).

#### Patients With Normal Complement Regulation

Twenty-one patients presented with normal complement regulation and coexisting hypertensive emergency (*n* = 12), kidney transplantation (*n* = 3), hemolysis, elevated liver enzymes, low platelets (HELLP; *n* = 3), drug-induced TMA (*n* = 2), and postsurgical TMA (*n* = 1).

Of these patients, 20 had follow-up data, with a median follow-up of 0.5 years (IQR 0.3–2.4 years) years (Table 1). Seven of 20 (35%) patients were treated with plasma therapy; eculizumab was initiated in 5 patients not responding to standard of care (Supplementary Table S5). Two kidney donor recipients were treated with immunosuppressive drugs (i.e., prophylactic treatment for rejection).

Nine of 20 (45%) patients achieved a renal response, either a CR (*n* = 5) or PR (*n* = 4). Eight remitted patients had been treated with standard of care only, contrasting patients with C-TMA and coexisting conditions (8/9 vs. 2/14, *P* < 0.001). ESKD developed in 9 patients, including 3 of 5 (60%) patients who had been treated with eculizumab. Of note, 1 patient who had received 1 dose of eculizumab had achieved a PR before drug administration. In contrast to patients with C-TMA, none of the patients experienced TMA recurrence (Supplementary Table S1), including 4 kidney donor recipients (Figures 2b and 2c).

Two patients died during follow-up; the causes of death were pancreatitis (*n* = 1) and unknown (*n* = 1).

### *Ex vivo* C5b9 Formation, Therapeutic Complement Inhibition, and TMA Recurrence

**Figure 3:**
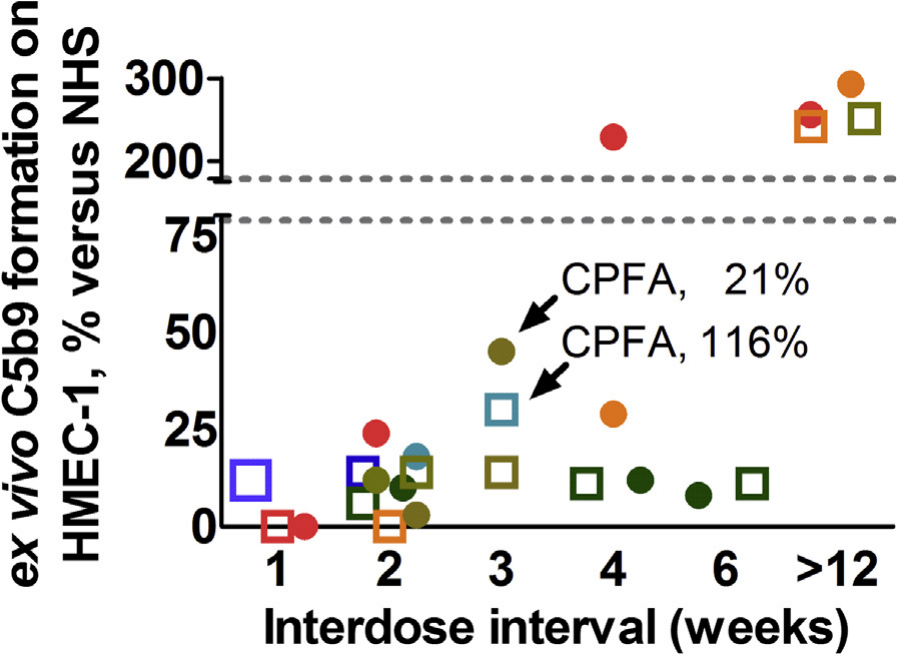
Prolonged interdose interval and *ex vivo* C5b9 formation on the perturbed endothelium in patients treated with eculizumab. *Ex vivo* C5b9 formation after incubation of perturbed endothelial cells with serum from patients treated with eculizumab using various interdose intervals, i.e., 1 week (*n* = 3; dose 900 mg), 2 weeks (*n* = 9; dose 1200 mg), 3 weeks (*n* = 3; dose 1200 mg), 4 weeks (*n* = 4; dose 1200 mg), or 6 weeks (*n* = 2; dose 1200 mg). Two patients with a prolonged interdose interval of 3 weeks and attenuated *ex vivo* C5b9 formation on the perturbed endothelium had a classical pathway functional activity (CPFA) above the recommended cutoff of 10% (Table 3). Also, sera from 4 patients not treated with eculizumab for ≥12 weeks obtained at the time of quiescent disease were tested (pathogenic variant in *CFI*, *n* = 1; pathogenic variant in *CFH*, *n* = 1; no genetic variants, *n* = 2); these samples induced massive *ex vivo* C5b9 formation on the perturbed endothelium, confirming the risk for unrestrained complement activation. Each patient has been denoted by a distinct symbol and color; dots tag patients with rare variants in complement genes. Normal range, *ex vivo* C5b9 formation of 78.78% to 178.62% compared with normal human serum (NHS). HMEC-1, human microvascular endothelial cell-1.

Follow-up samples from 14 patients with C-TMA treated with eculizumab (coexisting conditions, *n* = 11; primary aHUS, *n* = 3), including 6 patients who carried rare variants in complement genes, were available to test for *ex vivo* C5b9 formation. C5b9 formation on the perturbed endothelium was attenuated when incubated with samples from patients on eculizumab, confirming the assay’s specificity. We prolonged the interdose interval beyond 2 weeks and measured the classical pathway functional activity and *ex vivo* C5b9 formation on the perturbed endothelium in 7 cases (Figure 3, Table 3). Persistent inhibition of *ex vivo* C5b9 formation was achieved in 6 patients, including 2 patients with a classical pathway functional activity >10%. None of the patients experienced a relapse despite a prolonged interdose interval.

**Table 3:** Clinical parameters and complement measures in patients treated with eculizumab and a prolonged interdose interval

No.	Coexisting condition	Variant(s)	Interdose interval, weeks	Hb, mmol/l	LDH, U/l	Platelets, ×10^9^/L	Creatinine, μmol/l	C4, g/l	C3, g/l	CPFA, %	*Ex vivo* C5b9, % of the control^a^
M00018	None	C3, *CFI*	4	7.7	294	340	32	ND	ND	1	12
			6	8.2	237	299	38	0.19	1.12	2	8
M06518	Surgery	None	3	6.1	166	156	103	ND	ND	116	30
M06018	Hypertensive emergency	None	3	7.7	134	236	399	0.22	1.10	3	14
M99917	Hypertensive emergency	*CFI*, *THBD*	3	8.6	165	210	190	ND	ND	21	45
M08316	Pregnancy		4	8.7	139	204	106	0.22	0.82	1	11
			6	8.2	132	174	103	0.24	0.85	2	11
M01715	Hypertensive emergency	*CFI*	4	7.4	141	393	288	0.29	1.16	102	229
M04010	Hypertensive emergency	*CFH*	4	6.8	138	216	132	0.21	0.56	0	29

CPFA, classical pathway functional activity; Hb, hemoglobin; LDH, lactate dehydrogenase.

Case numbers are taken from the Maastricht cohort.

a *Ex vivo* C5b9 formation on the perturbed endothelium.

Four patients with TMA recurrence not treated with therapeutic complement inhibition were tested for *ex vivo* C5b9 formation. Patients invariably presented with massive *ex vivo* C5b9 formation on the resting endothelium, whereas samples from these patients obtained at the time of quiescent disease showed normal *ex vivo* C5b9 formation on the resting endothelium.

## Discussion

The recognition of complement dysregulation as the cause of TMA is important to select patients for therapeutic complement inhibition. Here, we show that complement dysregulation, defined by massive *ex vivo* C5b9 formation or pathogenic variants in complement genes, is prevalent in patients with TMA presenting with coexisting conditions and that these features are linked to poor kidney outcomes. Massive *ex vivo* C5b9 formation was associated with rare variants in complement genes and favorable renal response to therapeutic complement inhibition, confirming that these patients fall within the spectrum of C-TMA. Normal *ex vivo* C5b9 formation indicated an acute, nonrelapsing form of TMA.

Over the last decade, the approach of TMA has transformed and focused on the recognition of complement dysregulation in the earliest possible stage of disease.^7^^,^^8^ Notably, profound systemic hemolysis can be lacking in ≤60% patients with C-TMA and a kidney biopsy specimen may therefore need to be obtained to detect the TMA. Low C3 levels may suggest C-TMA at an early stage of disease, although routine complement measures are not specific for C-TMA.^12^^,^^28^ DNA sequencing is considered of high specificity but time-consuming, and therefore cannot be used to select patients for treatment. Here, we show that *ex vivo* C5b9 formation can categorize patients along the spectrum of TMA, including patients presenting with coexisting conditions. Most patients with massive *ex vivo* C5b9 formation who had been treated with eculizumab achieved a favorable renal response, whereas the prognosis of untreated patients was dire. Massive *ex vivo* C5b9 formation can therefore contribute to a rapid diagnosis of C-TMA and, moreover, may guide treatment decisions.

At the time of quiescent disease, *ex vivo* C5b9 formation normalized on the resting but not perturbed endothelium, indicating that the endothelium’s capacity to regulate complement normalized. In line with Galbusera *et al.*,^29^ TMA recurrence was associated with massive *ex vivo* C5b9 formation, similar to the first presentation, underscoring that the *ex vivo* test reflects the dynamic process of endothelium-restricted complement activation. In contrast, the modified Ham test using human endothelial hybrid cells that lack membrane-bound complement regulators (i.e., CD55 and CD59) cannot differentiate acute from quiescent disease.^30^ Moreover, serum samples from a subset of patients with HELLP show similar results compared with those from patients with C-TMA when using the modified Ham test.^31^ Indeed, secondary complement activation occurs in HELLP.^32^ The occurrence of HELLP in pregnant women treated with eculizumab,^33^ absence of pathogenic variants in complement genes,^31^ and favorable kidney survival plead against complement dysregulation.^34^ Thus, our test appears to be more specific than the modified Ham test for the detection of complement dysregulation. We advocate that our *ex vivo* test, when prospectively validated, can be implemented in routine clinical practice, although a specialized laboratory is needed to execute the test.

Rare variants in complement genes or FHAA confirmed the predisposition in about half the patients with massive *ex vivo* C5b9 formation on the resting endothelium, resembling primary aHUS.^3^ Our observation that patients with neither genetic variants nor FHAA may present with massive *ex vivo* C5b9 formation points to a circulating factor that affects complement regulation,^18^^,^^35^ either related to common variants in complement genes (i.e., minor allele frequency ≥0.1%) with *in vitro* studies showing functional consequences or a yet unidentified factor. TMA recurrence was common in patients with pathogenic variants, corroborating previous studies.^8^^,^^36^ Patients should therefore be screened for rare variants in complement genes to inform the long-term prognosis and therefore guide treatment decisions during follow-up.

The nomenclature on TMAs, considered to indicate targets for treatment, states that C-TMA should be reserved for patients not presenting with coexisting conditions.^7^^,^^8^ Three-quarters of our patients with C-TMA, however, presented with coexisting conditions. Many of such patients presented with typical features of TMA after review of a kidney biopsy specimen and coexisting conditions recently linked to a high prevalence of rare variants in complement genes—that is, hypertensive emergency,^37^, ^38^, ^39^ pregnancy,^40^ and de novo TMA after kidney transplantation^41^—whereas profound systemic hemolysis appeared uncommon. The prevalence of rare variants in complement genes is higher compared with controls (i.e., ∼5%).^42^ In contrast, rare variants in complement genes were not prevalent (i.e., ∼5% patients) in a French cohort of patients with TMA and coexisting drug use, autoimmunity, infections, and cancer among other causes.^42^ Pathogenic variants, identified in 2% of French patients, were associated with severe kidney involvement or TMA recurrence, similar to C-TMA. Therefore, the TMAs in the French cohort likely represent a mixture of distinct causes, only some of which may be linked to complement dysregulation.^19^ The phenotype of their cohort resembled our patients with normal *ex vivo* C5b9 formation: an acute, nonrelapsing form of TMA.

Observational studies showed conflicting results on the efficacy of eculizumab for the treatment of TMA presenting with coexisting conditions.^42^^,^^43^ Most responding patients presented with drug-induced TMA and mild-to-moderate kidney involvement.^43^ However, no studies have linked complement dysregulation to drug-induced TMA. The offending drug was stopped and the clinical response may therefore reflect the natural course of drug-induced TMA.^44^ Here, we show that most patients with C-TMA and coexisting conditions treated with therapeutic complement inhibition achieved a renal response, contrasting untreated patients. We advocate the use of therapeutic complement inhibition, either eculizumab or therapies under development, in selected patients presenting with coexisting conditions and, in particular, patients with massive *ex vivo* C5b9 formation. Nonresponding patients with massive *ex vivo* C5b9 formation presented with severe kidney disease and advanced chronicity scores on review of the kidney biopsy specimen. Future prospective trials, however, are needed to assess the efficacy of therapeutic complement inhibition in patients with TMA and coexisting conditions. The prognostic value of vascular damage, glomerulosclerosis, and interstitial fibrosis seen on review of a kidney biopsy specimen should also be studied.^45^

The optimal treatment regimen, that is, dosage and duration of eculizumab for the treatment of C-TMA, remains to be established. Eculizumab biweekly from the fifth week of treatment onward has been shown to block C5 activation as indicated by a classical pathway functional activity <10%. Eculizumab, however, can exceed the recommended target by up to 15-fold using the standard regimen.^46^ In selected cases, we show that *ex vivo* C5b9 formation can be attenuated when using a prolonged interdose interval. A prolonged interdose interval was associated with a classical pathway functional activity above the recommended cutoff in some patients, corroborating previous observations.^29^ None of the patients experienced a relapse, suggesting that trough levels below the recommended target may prevent TMA recurrence and potentiate lower costs of treatment.

In conclusion, patients with TMA and coexisting conditions may present with C-TMA and should therefore be screened for *ex vivo* C5b9 formation and rare variants in complement genes to categorize the TMA. The risk of ESKD appeared lower in patients with massive *ex vivo* C5b9 formation at presentation and treated with eculizumab. We therefore advocate that *ex vivo* C5b9 formation may be used to select patients for therapeutic complement inhibition, whilst rare variants in complement genes inform the long-term prognosis. Prospective studies are needed to test the hypothesis that therapeutic complement inhibition can improve the outcome of such patients.

## Disclosure

JM is supported by the Fonds National pour la Recherche Scientifique, the Fondation Saint-Luc, the Fonds de Recherche Clinique des Cliniques universitaires Saint-Luc, the Association pour l’Information et la Recherche sur les maladies rénales Génétiques (AIRG Belgium), and a research grant from Alexion Pharmaceuticals. All the other authors declared no competing interests.

## Supplementary Material

**Supplementary Methods**

**Figure S1.** Flowchart.

**Table S1.** Baseline characteristics according to the atypical hemolytic uremic syndrome type classification.

**Table S2.** *Ex vivo* C5b9 formation on the perturbed endothelium when using serum samples from disease controls showed a specificity of 95%.

**Table S3.** Baseline characteristics of patients with complement-mediated thrombotic microangiopathy and coexisting conditions treated with eculizumab or not.

**Table S4.** Indications for immunosuppressive agents.

**Table S5.** Clinical characteristics of patients with TMA and normal complement regulation treated with eculizumab.

**STROBE Statement**
