# Supplementary material for: Functional and Genetic Landscape of Complement Dysregulation Along the Spectrum of Thrombotic Microangiopathy and its Potential Implications on Clinical Outcomes
Source: Kidney Int Rep. 2021 Feb 3;6(4):1099–109. doi: 10.1016/j.ekir.2021.01.034 (PMC8071658; doi:10.1016/j.ekir.2021.01.034)
Supplement: Supplementary File (PDF) [file mmc1.pdf]

Supplementary methods. Patients with thrombotic microangiopathy (TMA) were classified according to the current nomenclature.(1) The following definitions have been used:

Antiphospholipid syndrome-related TMA. Patients with TMA and persistent phospholipid serum reactivity (i.e., lupus anticoagulant, anti-β2 glycoprotein I IgG, and/or anti-cardiolipin IgM/IgG).(2)

De novo TMA after kidney transplantation. Donor kidney recipients with TMA unrelated to calcineurin nephrotoxicity, infection, and antibody-mediated rejection (i.e., neither donor specific alloantibodies nor C4d deposits along the peritubular capillaries on allograft biopsy) who presented with end-stage kidney disease not related to TMA in their native kidneys.

Drug-induced TMA. TMA related to drugs reported in the Oklahoma Registry and Blood Center of Wisconsin to have a definite causal association with TMA (the data can be found on: https://www.ouhsc.edu/platelets/DITMA.htm).(3)

HELLP (hemolysis, elevated liver enzymes, and low platelets). Patients with the onset of TMA during pregnancy, aspartate and/or alanine aminotransferase at least 2 times the upper limit of normal, and clinical improvement after delivery.

Hypertensive emergency. Patients with TMA presenting with typical pathologic features of severe hypertension (i.e., myxoid intimal changes, hypertrophy of the arterial vessel walls, and/or fibrinoid necrosis of arterioles) on kidney biopsy, severe hypertension (i.e., blood pressure levels of at least 180 mmHg systolic and/or 120 mmHg diastolic), and evidence of impending or progressive target organ damage outside the kidneys.(4, 5)

Postsurgical TMA. Patients with the onset of TMA within 30 days after surgery.(6)

Pregnancy-associated atypical hemolytic uremic syndrome. Patients with the onset of TMA during pregnancy or within the first 12 weeks postpartum.(7)

Primary atypical hemolytic uremic syndrome. Patients with TMA not presenting with coexisting conditions.

Shiga toxin-producing E. coli hemolytic uremic syndrome. TMA related to Shiga toxin-producing E. *coli* infection.

Streptococcal hemolytic uremic syndrome. TMA related to S. *pneumoniae* infection.

Thrombotic thrombocytopenic purpura. Patients with TMA presenting with an enzymatic activity of von Willebrand factor cleaving protease <10% as based on FRETS-VWF73 assay and/or platelets $<30 \times 10^9$/L and serum creatinine ≤200 µmol/L.(8)

Figure S1. Flowchart. Three patients with pathogenic variants in complement genes were classified as complement-mediated (C-)TMA, although no baseline serum sample was available to test for *ex vivo* C5b9 formation on the endothelium.

aHUS, atypical hemolytic uremic syndrome. APS, antiphospholipid syndrome. C-TMA, complement-mediated thrombotic microangiopathy. HUS, hemolytic uremic syndrome. TMA, thrombotic microangiopathy. TTP, thrombotic thrombocytopenic purpura.

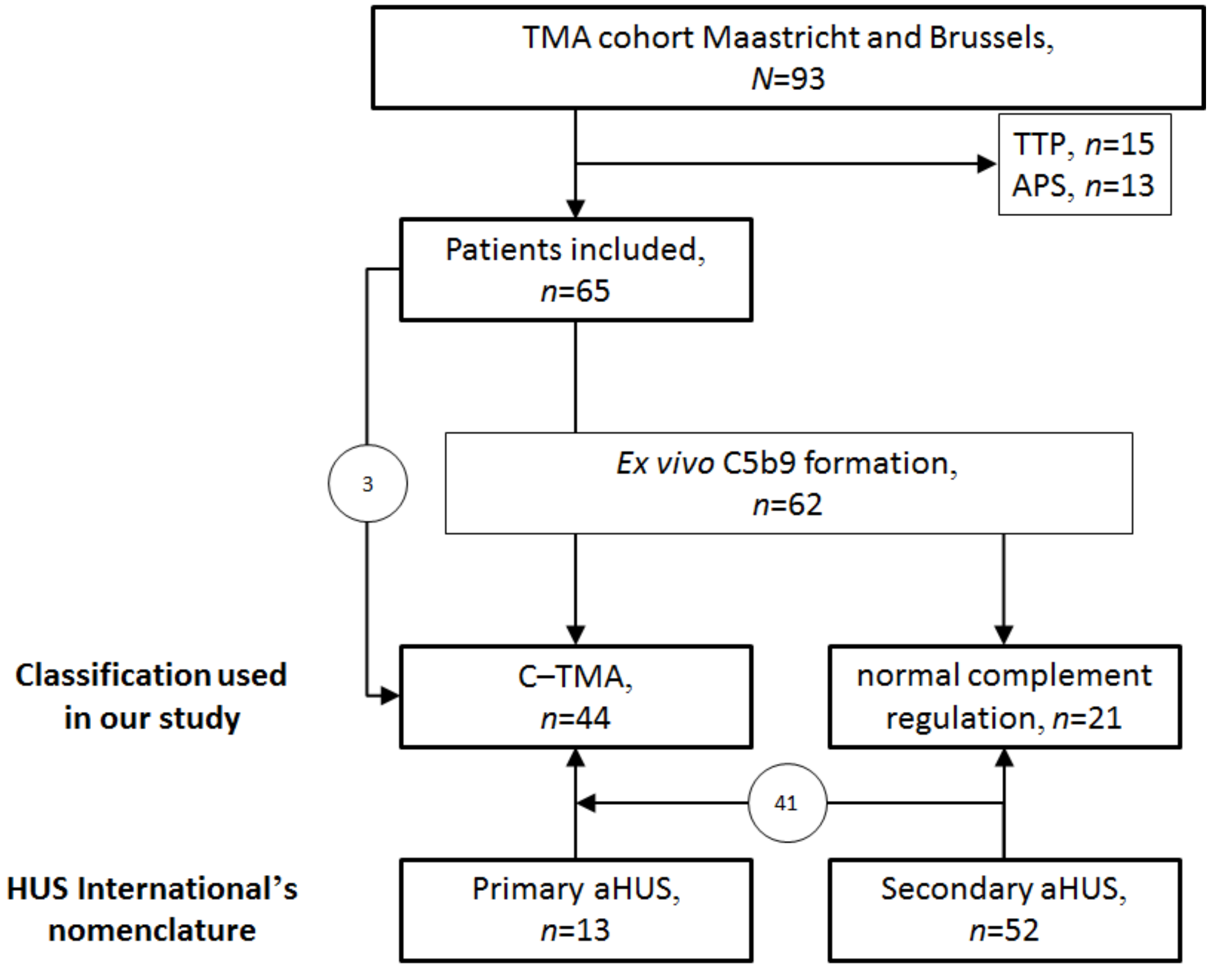

Table S1. Baseline characteristics according to the atypical hemolytic uremic syndrome type classification.(1)

	Primary aHUS	Secondary aHUS	
C-TMA	Yes	Yes	No
**HUS International's nomenclature**(1)			
Coexisting condition(s), *n*/*N*	0/13	31/31[b]	21/21[b]
Hypertensive emergency	0	18[b]	12[b]
Pregnancy	0	8	0[c]
TMA after kidney transplantation	0	2	3
Postsurgical TMA	0	2	1
Streptococcal HUS	0	1	0
HELLP	0	0	3
Drug-induced TMA	0	0	2
**Features at presentation**			
M/F	5/8	14/17	12/9
European (%)	12 (92)	31 (100)	16 (76)
Age, years	30±25	38±13	42±13
Creatinine, µmol/L	321 (193-407)	561 (356-1,065)[b]	485 (231-778)
Dialysis (%)	5 (38)	22 (71)	11 (52)
Hemolysis (%)	12 (92)	13 (42)[b]	11 (52)[a]
Systemic hemolysis (%)	9 (69)	9 (29)[a]	8 (38)
Platelets, $\times 10^9$/L	36 (12-200)	133 (75-228)[a]	95 (52-178)
LDH, U/L	1,251 (711-2,390)	680 (305-1,486)[a]	762 (465-1,222)[a]
ADAMTS13's activity >10%, *n*/*N*	10/10	21/21	17/17
Low C4, *n*/*N*	0/10	5/29	0/18
Low C3, *n*/*N*	6/11	12/30	1/18[b, c]
Massive *ex vivo* C5b9 formation, *n*/*N*	11/11	30/30	0/21[b, d]
Rare variant(s)/FHAA (%)	9 (70)	11 (35)	0 (0)[b, d]
Pathogenic (%)	6 (46)	11 (35)	0 (0)[b, d]
Combined variants	1	1	0
*MCP*ggaac, *n*/*N*	7/11	9/20	12/19
**Treatment**			
Plasma therapy (%)	10 (77)	21 (68)	7 (33)[b, c]
Immunosuppression (%)	1 (8)	11 (35)	2 (10)
Eculizumab (%)	5 (38)	14 (45)	5 (26)
Days after diagnosis, median	4 (range, 1-19)	7 (range, 1-100)	4 (range, 2-37)
Doses, median	10 (range, 2-21)	14 (range, 4-70)	4 (range, 1-10)
Ongoing, *n*/*N*	1/5	2/14	0/5
**Clinical outcome**			
Patients, *n*/*N*	13/13	30/31	20/21
Follow-up, years	2.1 (1.0-9.1)	2.3 (0.7-6.5)	0.5 (0.3-2.4)[a, d]
Renal response (%)	10 (77)	14 (47)	9 (45)
Complete remission	9	6	4
Partial remission	1	8	5
ESKD at 3 months (%)	2 (15)	15 (50)[a]	7 (35)
ESKD at last follow-up (%)	3 (23)	16 (53)	8 (45)
Patients with TMA recurrence (%)	3 (23)	8 (27)	0[a, c]
Deceased at 3 months (%)	0	1 (3)	1 (5)
Deceased at last follow-up (%)	0	3 (10)	2 (10)

[a]$P \leq 0.05$ and [b]$P < 0.01$ versus primary aHUS.

[c]$P \leq 0.05$ and [d]$P < 0.01$ versus C-TMA and coexisting conditions.

Table S2. *Ex vivo* C5b9 formation on the perturbed endothelium when using serum samples from disease controls showed a specificity of 95%. Patients' samples have been obtained at the time of presentation prior to treatment.

	N	Massive *ex vivo* C5b9 formation
**Thrombotic microangiopathy**		
TTP	7	0
APS-related	7	1
STEC-HUS	1	0
**Glomerulopathies**		
C3G	10	1
APS nephropathy*	3	0
AGN	2	0
**Hypertension**		
Arterionephrosclerosis	5	0
Hypertensive emergency†	4	0

*Focal cortical necrosis without morphologic features of thrombotic microangiopathy on kidney biopsy. †Patients presenting with hypertensive emergency and an estimated glomerular filtration rate >45 mL/min/1.73m^2.

AGN, ANCA-associated glomerulonephritis. APS, antiphospholipid syndrome. C3G, C3 glomerulopathy. STEC-HUS, Shiga toxin producing E. *coli*-associated hemolytic uremic syndrome. TTP, thrombotic thrombocytopenic purpura.

Table S3. Baseline characteristics of patients with complement-mediated thrombotic microangiopathy and coexisting conditions treated with eculizumab or not.

	**Eculizumab**	**Untreated**	***P* value**
Patients	14	16	
**Coexisting conditions**			
Hypertensive emergency	6	11	0.3
Pregnancy	4	4	1.0
De novo after kidney transplantation	1	1	1.0
Miscellaneous	3	0	0.09
**Features at presentation**			
M/F	6/8	7/9	1.0
Caucasian (%)	14 (100)	16 (100)	1.0
Age, years	39 (29-56)	33 (28-38)	0.2
Creatinine, µmol/L	492 (345-583)	854 (566-1,181)	0.01
Dialysis (%)	8 (57)	14 (88)	0.1
Hemolysis (%)	6 (43)	6 (38)	1.0
Platelets, $\times 10^9$/L	90 (46-277)	138 (95-204)	0.8
LDH, U/L	620 (304-1,098)	867 (312-2,044)	0.6
Low C4, *n*/*N*	4/13	1/15	0.2
Low C3, *n*/*N*	5/13	7/16	1.0
Rare variant(s)/FHAA (%)	5 (36)	8 (50)	0.5
Pathogenic (%)	3 (21)	8 (50)	0.1
Combined variants	2	1	0.6
*MCP*ggaac, *n*/*N*	5/11	4/8	1.0
**Treatment**			
Plasma therapy (%)	12 (86)	9 (56)	0.1
Immunosuppression (%)	6 (42)	5 (31)	0.7
Eculizumab (%)	14 (100)	–	
Days after diagnosis, median	6 (range, 0-100)	–	
Doses, median	14 (9-24)	–	
Ongoing, *n*/*N*	2/14	–	

F, female. FHAA, factor H autoantibodies. M, male.

Table S4. Indications for immunosuppressive agents.

No.	Age, y./ gender	Coexisting condition	Creatinine, µmol/L	Hemolysis	Platelets, $\times 10^9$/L	Immunosuppressive agent(s)	Ecu	Indication	Outcome
**Complement-mediated thrombotic microangiopathy**									
M00016	4/M	–	311	+	28	CS	–	DEAP-HUS	CR
M06018	26/M	HE	805	–	171	CS	+	–	PR
M01416	72/F	HE	356	+	75	CS, MMF	+	acute TIN	PR
M02715	28/F	HE	1,065	–	228	CS, MMF	–	–	ESKD
M01715	41/F	HE	334	–	291	CS, MMF	+	–	PR
M04010	32/F	HE	1,138	+	142	CS	–	–	ESKD
M03307	37/M	HE	586	+	100	CS	–	–	ESKD
M00503	32/F	P-aHUS	1,388	+	212	CS	–	–	ESKD
M06019	30/F	P-aHUS	411	+	36	CS	+	–	CR
M06518	74/F	Surgery	220	+	24	CS	+	–	PR
B07	35/M	Kidney donor	519	–	93	TAC, MMF, CS	–	Prophylaxis*	ESKD
B33	24/M	Kidney donor	309	–	252	TAC, MMF, CS	+	Prophylaxis*	PR
**Normal complement regulation**									
M00018	54/F	Kidney donor	242	–	36	CS	–	Prophylaxis*	PR
B10	52/M	Kidney donor	795	–	69	TAC, CS	–	Prophylaxis*	ESKD

*Prophylactic treatment for rejection or graft versus host disease.

aHUS, atypical hemolytic uremic syndrome. B, Brussels' cohort. CR, complete renal remission. CS, corticosteroids. DEAP-HUS, deficiency of CFHR and autoantibody-positive hemolytic uremic syndrome. Ecu, eculizumab. ESKD, end-stage kidney disease. HE, hypertensive emergency. M, Maastricht' cohort. MMF, mycophenolate mofetil. P-aHUS, pregnancy-associated aHUS. PR, partial renal remission. RTX, rituximab. SLE, systemic lupus erythematosus. TAC, tacrolimus. TIN, tubulointerstitial nephritis.

Table S5. Clinical characteristics of patients with TMA and normal complement regulation treated with eculizumab.

No.	Sex/ age, y.	Coexisting condition	Creatinine, µmol/L	Hemolysis	Platelets, $\times10^9$/L	GS, *n*/*N*	IF/TA, %	Dialysis	Treatment	Time to Ecu (d)	Ecu doses	Outcome
M13519	M/38	HE	984	–	83	3/13	20	+	BP, PLEX	4	4	ESKD
M09419	M/63	HE	546	–	122	1/7	40	+	BP, PLEX	2	2	ESKD
M11818	M/37	Surgery	626	+	56	0/14	<5	+	PLEX	3	10	CR
M01217	M/38	HE	726	+	62	5/21	40	+	BP, PLEX	7	5	ESKD, died
B03	M/41	HE	1,017	+	95	3/14	15	+	BP, PLEX	37	1	PR*

B, Brussels' cohort. CR, complete renal remission. Ecu, eculizumab. ESKD, end-stage kidney disease. GS, glomerulosclerosis. HE, hypertensive emergency. IF/TA, interstitial fibrosis/tubular atrophy. M, Maastricht' cohort. PR, partial renal remission.

*PR was achieved prior to administration of eculizumab.

References

1. Loirat C, Fakhouri F, Ariceta G, Besbas N, Bitzan M, Bjerre A, et al. An international consensus approach to the management of atypical hemolytic uremic syndrome in children. Pediatr Nephrol. 2016;31(1):15-39.
2. Miyakis S, Lockshin MD, Atsumi T, Branch DW, Brey RL, Cervera R, et al. International consensus statement on an update of the classification criteria for definite antiphospholipid syndrome (APS). J Thromb Haemost. 2006;4(2):295-306.
3. Saleem R, Reese JA, George JN. Drug-induced thrombotic microangiopathy: An updated systematic review, 2014-2018. Am J Hematol. 2018;93(9):E241-E3.
4. Timmermans S, Werion A, Damoiseaux J, Morelle J, Reutelingsperger CP, van Paassen P. Diagnostic and Risk Factors for Complement Defects in Hypertensive Emergency and Thrombotic Microangiopathy. Hypertension. 2020;75(2):422-30.
5. Cremer A, Amraoui F, Lip GY, Morales E, Rubin S, Segura J, et al. From malignant hypertension to hypertension-MOD: a modern definition for an old but still dangerous emergency. J Hum Hypertens. 2016;30(8):463-6.
6. Sridharan M, Hook CC, Leung N, Winters JL, Go RS, Mayo Clinic Complement Alternative Pathway-Thrombotic Microangiopathy Disease-Oriented G. Postsurgical thrombotic microangiopathy: Case series and review of the literature. Eur J Haematol. 2019;103(4):307-18.
7. Bruel A, Kavanagh D, Noris M, Delmas Y, Wong EKS, Bresin E, et al. Hemolytic Uremic Syndrome in Pregnancy and Postpartum. Clin J Am Soc Nephrol. 2017;12(8):1237-47.
8. Coppo P, Schwarzinger M, Buffet M, Wynckel A, Clabault K, Presne C, et al. Predictive features of severe acquired ADAMTS13 deficiency in idiopathic thrombotic microangiopathies: the French TMA reference center experience. PLoS One. 2010;5(4):e10208.

Modified STROBE Statement—checklist of items that should be included in reports of observational studies (Cohort/Cross-sectional and case-control studies)

	Item No	Recommendation
**Title and abstract**	1 Yes	(*a*) Indicate the study's design with a commonly used term in the title or the abstract
		(*b*) Provide in the abstract an informative and balanced summary of what was done and what was found
**Introduction**		
Background/rationale	2 Yes	Explain the scientific background and rationale for the investigation being reported
Objectives	3 Yes	State specific objectives, including any prespecified hypotheses
**Methods**		
Study design	4 Yes	Present key elements of study design early in the paper
Setting	5 Yes	Describe the setting, locations, and relevant dates, including periods of recruitment, exposure, follow-up, and data collection
Participants	6 Yes	(*a*) *Cohort study*—Give the eligibility criteria, and the sources and methods of selection of participants. Describe methods of follow-up<br>~~*Case-control study*—Give the eligibility criteria, and the sources and methods of case ascertainment and control selection. Give the rationale for the choice of cases and controls~~<br>*Cross-sectional study*—Give the eligibility criteria, and the sources and methods of selection of participants
Variables	7 Yes	Clearly define all outcomes, exposures, predictors, potential confounders, and effect modifiers. Give diagnostic criteria, if applicable
Data sources/ measurement	8* Yes	For each variable of interest, give sources of data and details of methods of assessment (measurement).
Bias	9 Yes	Describe any efforts to address potential sources of bias
Study size	10 N/a.	Explain how the study size was arrived at (if applicable)
Quantitative variables	11 Yes	Explain how quantitative variables were handled in the analyses. If applicable, describe which groupings were chosen and why
Statistical methods	12 Yes	(*a*) Describe all statistical methods, including those used to control for confounding

		(*b*) Describe any methods used to examine subgroups and interactions
		(*c*) Explain how missing data were addressed
		(*d*) *Cohort study*—If applicable, explain how loss to follow-up was addressed<br><br>~~*Case-control study*—If applicable, explain how matching of cases and controls was addressed~~<br><br>~~*Cross-sectional study*—If applicable, describe analytical methods taking account of sampling strategy~~
		(*e*) Describe any sensitivity analyses
**Results**		
Participants	13* Yes	(a) Report numbers of individuals at each stage of study—eg numbers potentially eligible, examined for eligibility, confirmed eligible, included in the study, completing follow-up, and analyzed
		(c) **Use of a flow diagram**
Descriptive data	14* Yes	(a) Give characteristics of study participants (eg demographic, clinical, social) and information on exposures and potential confounders
		(b) Indicate number of participants with missing data for each variable of interest
		(c) *Cohort study*—Summarise follow-up time (eg, average and total amount)
Outcome data	15* Yes	*Cohort study*—Report numbers of outcome events or summary measures over time
		~~*Case-control study*—Report numbers in each exposure category, or summary measures of exposure~~
		Cross-sectional study—Report numbers of outcome events or summary measures
Main results	16 Yes	(*a*) Give unadjusted estimates and, if applicable, confounder-adjusted estimates and their precision (eg, 95% confidence interval). Make clear which confounders were adjusted for and why they were included
Other analyses	17 Yes	Report other analyses done—eg analyses of subgroups and interactions, and sensitivity analyses
**Discussion**		

Key results	18	Yes	Summarise key results with reference to study objectives
Limitations	19	Yes	Discuss limitations of the study, taking into account sources of potential bias or imprecision. Discuss both direction and magnitude of any potential bias
Interpretation	20	Yes	Give a cautious overall interpretation of results considering objectives, limitations, multiplicity of analyses, results from similar studies, and other relevant evidence
Generalisability	21	Yes	Discuss the generalisability (external validity) of the study results

*Give information separately for cases and controls in case-control studies and, if applicable, for exposed and unexposed groups in cohort and cross-sectional studies.

Note: An Explanation and Elaboration article discusses each checklist item and gives methodological background and published examples of transparent reporting. The STROBE checklist is best used in conjunction with this article (freely available on the Web sites of PLoS Medicine at http://www.plosmedicine.org/, Annals of Internal Medicine at http://www.annals.org/, and Epidemiology at http://www.epidem.com/). Information on the STROBE Initiative is available at www.strobe-statement.org.
